# Supplementary material for: Alterations of Plasma Pro-Inflammatory Cytokine Levels in Children with Refractory Epilepsies
Source: Children (Basel). 2022 Oct 1;9(10):1506. doi: 10.3390/children9101506 (PMC9600205; doi:10.3390/children9101506)
Supplement: Supplementary file 1 [file children-09-01506-s001.zip › children-1900690-supplementary.pdf]

Table S1. Correlation between age and CCL11 Levels.

Correlations

			CCL11	age
Spearman's rho	CCL11	Correlation Coefficient	1.000	-0.126
		Sig. (2-tailed)	.	0.356
		N	56	56
	age	Correlation Coefficient	-0.126	1.000
		Sig. (2-tailed)	0.356	.
		N	56	56

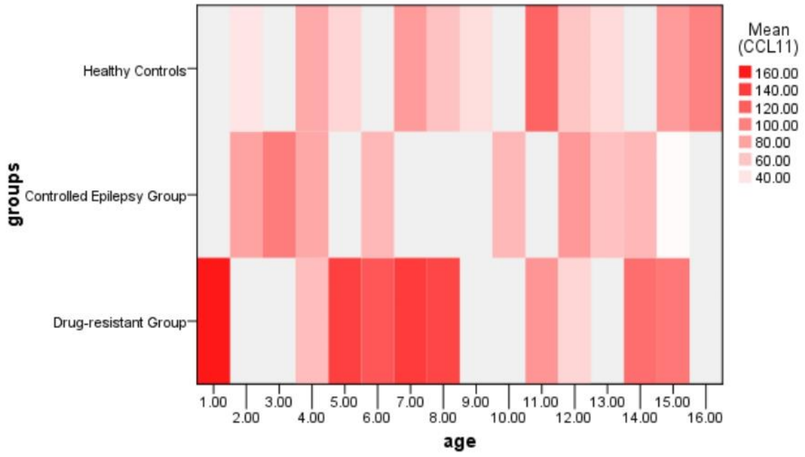

Figure S1

A heat map representation of the Spearman correlation matrix for CCL11 and age correlation
